# Fetal programming under maternal heat stress: a focus on skeletal muscle growth and nutrition in livestock

**DOI:** 10.1017/S2040174425100111

**Published:** 2025-08-01

**Authors:** Weicheng Zhao, Rosa I. Luna Ramirez, Robert P. Rhoads, Laura D. Brown, Sean W. Limesand

**Affiliations:** 1 School of Animal and Comparative Biomedical Sciences, University of Arizona, Tucson, AZ, USA; 2 School of Animal Sciences, Virginia Tech, Blacksburg, VA, USA; 3 Department of Pediatrics, University of Colorado Anschutz Medical Campus, Aurora, CO, USA

**Keywords:** Developmental programming, hyperthermia, intrauterine growth restriction, livestock, pregnancy

## Abstract

An adverse *in utero* experience negatively impacts perinatal growth in livestock. Maternal heat stress (HS) during gestation reduces placental growth and function. This progressive placental insufficiency ultimately leads to fetal growth restriction (FGR). Studies in chronically catheterized fetal sheep have shown that FGR fetuses exhibit hypoxemia, hypoglycemia, and lower anabolic hormone concentrations. Under hypoxic stress and nutrient deficiency, fetuses prioritize basal metabolic requirements over tissue accretion to support survival. Skeletal muscle is particularly vulnerable to HS-induced placental insufficiency due to its high energy demands and large contribution to total body mass. In FGR fetuses, skeletal muscle growth is reduced, evidenced by smaller myofiber size and mass, reduced satellite cell proliferation, and slower rate of protein synthesis. Disruptions in skeletal muscle growth are associated with mitochondrial dysfunction, including reduced pyruvate flux into the mitochondrial matrix and lower complex I activity in the mitochondrial electron transport chain. This review summarizes current research on the mechanisms by which HS-induced placental insufficiency affects skeletal muscle growth in the fetus, with an emphasis on myogenesis, hypertrophy, protein synthesis, and energy metabolism. The evidence presented is primarily drawn from experiments using chronically catheterized fetal sheep exposed to maternal HS during mid-gestation. Additionally, we explore emerging nutritional strategies aimed at enhancing skeletal muscle growth in animals with FGR. These strategies hold promise not only for improving reproductive efficiency in livestock affected by prenatal stress but also for their translational relevance to human pregnancies complicated by placental insufficiency.

## Introduction

Climate change and the increasing frequency of extreme heat events present an emerging threat to mammalian reproductive health. Epidemiology studies in humans have shown that maternal exposure to sustained high temperatures during pregnancy is associated with an increased risk of preterm birth, perinatal morbidity, and mortality.^1–3^ In livestock species, such as sheep, cows, and pigs, environmental heat stress (HS) during gestation reduces embryo survival, fetal growth, and birth weight and increases perinatal mortality.^4–7^ Maternal HS also causes long-lasting effects on postnatal growth and metabolism. Ruminant offspring born to HS dams exhibited lower body weights in early postnatal stages.^8–12^ Furthermore, HS offspring developed a compromised passive immune function, altered body composition, lower fecundity, and increased rates of congenital abnormalities across livestock species.^8,12–16^ These physiological and developmental disruptions ultimately impact animal welfare and productive performance, leading to long-term economic losses for animal agriculture.^17–19^

**Figure 1.**
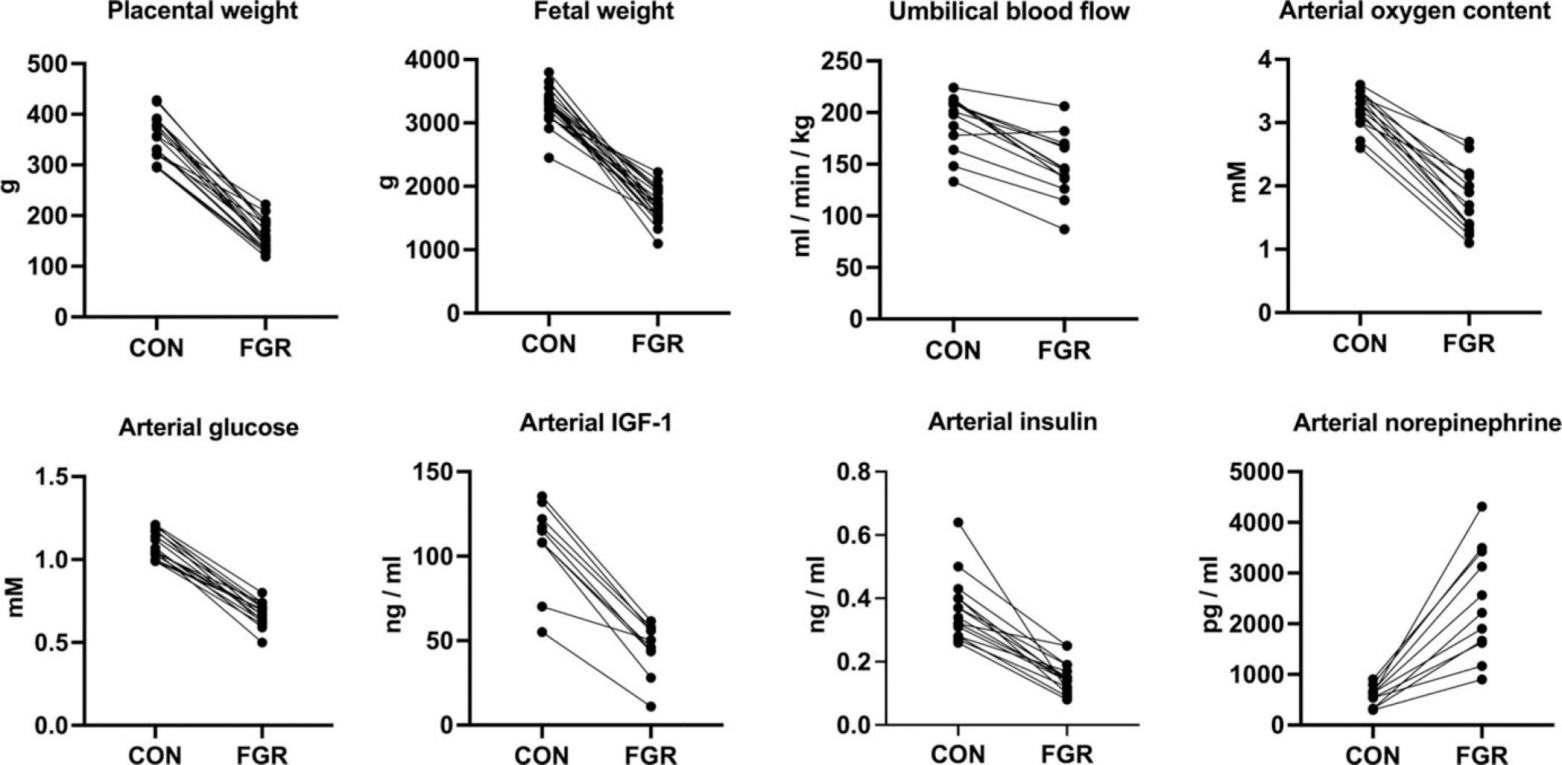
The impact of maternal heat stress-induced placental insufficiency on fetal and placental characteristics. Placental insufficiency and fetal growth restriction (FGR) were created by exposing pregnant ewes to elevated environmental temperatures (35–40°C; temperature-humidity index (THI): 83 to 89) during mid-gestation for 50–80 d, whereas control (CON) ewes were maintained under thermoneutral conditions (constant 25°C; THI: 71). Samples were collected and analyzed after heat exposure at approximately 130 d of gestation (dGA; term 149 dGA). Data were presented as group means as reported in.^21–40^ The lines connect CON and FGR group means from each study.

Placental insufficiency is a causative factor that programs the altered phenotypes seen in fetuses from HS dams. In mammals, placental growth precedes fetal growth, and perturbations during key periods of placental development can lead to placental insufficiency and, later in pregnancy, fetal growth restriction (FGR). Maternal strain from chronic environmental HS begins to affect placental growth as early as mid-gestation prior to any discernible effect on fetal growth in sheep.^20^ By using a well-established HS model of placental insufficiency in pregnant ewes, studies have shown that exposing pregnant ewes to cyclic HS (35 to 40°C; temperature-humidity index: 83–89) between mid and late gestation, coinciding with critical periods of placental development, results in a ≥40% reduction in placental and fetal weights near term (Fig. 1). FGR fetuses from HS-induced placental insufficiency also exhibit slower umbilical blood flow and lower anabolic substrate availability and develop hypoglycemia and hypoxemia (Fig. 1). Notably, these outcomes mirror the symptoms observed in human fetuses with FGR and represent a viable animal model system for studying developmental programming in FGR fetuses with placental insufficiency.

FGR is evident in skeletal muscle, which serves as the largest soft tissue by mass in the fetus and accounts for a majority of whole-body insulin-stimulated glucose uptake.^41,42^ The mechanisms underlying impaired muscle growth in HS-induced FGR fetuses are not fully understood but are likely driven by a combination of insufficient nutrient supply, reduced oxygen availability, and altered metabolism under hypoxic stress. If placental insufficiency begins early in pregnancy, the process of myogenesis (myofiber formation) is at risk. Fewer myofibers at an early stage may reduce the overall number of muscle fibers, which subsequently undergo hypertrophy (an increase in muscle fiber size). This reduction could potentially limit overall muscle mass and protein synthesis in the fetus. Importantly, reduced fetal skeletal muscle growth is not fully offset after birth because less lean tissue and greater fat deposition were observed in adult pigs^13,14^ and juvenile lambs^12^ born to HS dams.

This review focuses on our current understanding of how placental insufficiency affects fetal skeletal muscle growth, drawing primarily from research conducted on chronically catheterized fetal sheep with maternal HS-induced placental insufficiency. These studies provide insights into the mechanisms by which HS-induced placental insufficiency reduces nutrient and oxygen supply from the placenta and impairs hindlimb muscle fiber formation, hypertrophy, protein synthesis, and energy metabolism within mitochondria. Additionally, we explore recent advances in nutritional strategies designed to mitigate the negative impacts on skeletal muscle development in FGR animals. By summarizing these interventions, we aim to underscore their potential to enhance livestock performances and offer translational insights into targeting FGR-related complications in human health.

### Heat stress impact on placental growth and function

In sheep, the maximum placenta growth rate occurs between days 40 and 80 of gestational age (dGA), during which the placenta expands its surface area to form functional placentomes for maternal-fetal exchange.^43^ Reductions in placental growth and development were observed in mid-gestating ewes (75 dGA) following 25 d of maternal HS, even in the absence of reductions to fetal weight at this stage.^20^ The mechanisms by which HS restricts placental growth in livestock remain unclear. However, it is likely associated with a combination of physiological, vascular, hormonal, and cellular mechanisms that disrupt placental function and development. In HS animals, thermoregulatory adaptations redirect blood flow toward peripheral tissue, such as the skin, to enhance radiant heat loss.^44^ This adaptation is likely to reduce blood perfusion and cause cellular stress in visceral organs, including the placenta. It is well established that HS-induced placental growth restriction is associated with decreases in uterine and umbilical blood flows in late pregnancy.^21^

In mid-gestating ewes, placental growth restriction due to HS initially results in a compensatory increase in VEGF mRNA expression in the cotyledon after 15–20 d of HS.^45^ However, prolonged exposure to HS from mid to late gestation results in reduced mRNA expression of VEGF and its receptor in the cotyledon,^24^ as well as lower cotyledonary or umbilical expression of endothelial nitric oxide synthase.^46–48^ In addition to vascular impairment, HS is linked to lower maternal concentrations of progesterone and ovine placental lactogen, indicating adverse effects on the development and function of trophoblast cells responsible for steroid production and metabolism.^49,50^ Additionally, HS has been associated with cellular-level changes, such as reduced placental cell numbers, lower DNA content, and decreased protein synthesis capacity in pregnant ewes.^20,51^ These findings highlight that maternal HS disrupts placental growth and function through a multifactorial mechanism. However, it remains unclear whether these maladaptive changes provoke placental growth restriction or are simply a consequence of reduced placental mass in HS pregnant ewes.

The transport of oxygen, glucose, and amino acids across the placenta is mediated by passive diffusion, facilitated diffusion, and active transport, respectively.^52^ Placental transport capacity for oxygen and glucose is reduced in HS ewes during late gestation, as a result of decreased placental permeability.^21,22,53^ Additionally, HS ewes with placental insufficiency exhibit approximately 40% lower transplacental flux of amino acids from the maternal circulation to the fetus when adjusted for placental mass.^23,40^ Although reduced blood flow can limit placental uptake and transfer of flow-limited substrates such as oxygen, it is unlikely to be the primary factor limiting glucose and amino acid transport in HS ewes.^54^ Rather, impaired placental transport capacity for glucose and amino acids is likely due to a reduced surface area and lower abundance of nutrient transporters per unit of placental weight, as both nutrients are transported via specific transporter proteins on placental membranes.^55,56^ In HS ewes, expression of the glucose transporter 8 (GLUT 8) is decreased during late gestation.^25^ Recent transcriptomic analyses in HS pregnant pigs have also revealed widespread downregulation of major placental nutrient transporter mRNA expression by mid-gestation.^57^

### Fetal metabolic and endocrine response to heat stress-induced placental insufficiency

The developing fetus utilizes nutrients taken up from the umbilical circulation to meet two primary needs: supporting essential metabolic processes via oxidative metabolism and promoting tissue growth and protein accretion. In HS-induced FGR fetal sheep, there were lower weight-specific net fetal uptake rates for glucose,^25,38,39^ amino acids^53,58^ and oxygen despite an increased transplacental oxygen and glucose gradients.^28,39,58^ To explore the metabolic efficiency of fetal substrate utilization, the substrate/oxygen quotient was calculated (defined as the theoretical fraction of total fetal O_2_ consumption required to completely oxidize the net umbilical uptake of a particular substrate). Combined substrate/oxygen quotients for glucose, lactate, and amino acids were reduced to levels that barely exceed basic energy requirements for oxidative metabolism in whole sheep fetuses^53^ or in the hindlimb,^35^ which leaves little room for tissue accretion. This limited surplus of nutrients for energy reserves results in a 22% reduction in hindlimb linear growth rate in FGR fetuses compared to control fetuses.^35^

Fetal adaptation to systemic hypoxemia involves activation of the sympathetic nervous systems.^59^ Sympathetic overactivity is maintained by adrenergic-mediated hormones, such as catecholamines. A negative association between blood oxygen content and plasma noradrenaline concentrations was observed in FGR fetal sheep.^30,60,61^ Higher concentrations of plasma noradrenaline inhibit insulin secretion via the α2-adrenergic receptors on β-cells.^62^ Moreover, chronic exposure to elevated catecholamines persistently inhibits β-cell function and reduces both basal and glucose-stimulated insulin secretion (GSIS) in FGR fetuses.^39,60,63^ In addition to catecholamine-mediated inhibition of insulin secretion, low glucose and oxygen concentrations also decrease β-cell proliferation and insulin secretion.^61,64^ Together, these actions lower fetal insulin concentrations and limit fetal glucose uptake by peripheral tissues, which will conserve glucose for critical organs (e.g., brain and heart) under hypoglycemic conditions.^65^ We have demonstrated that surgical ablation of the fetal adrenal medulla prevents acute hypoxia-induced norepinephrine secretion in FGR fetal sheep and partially restores β-cell function and insulin secretion responsiveness.^37,66^ These findings highlight the role of adrenergic signaling in regulating fetal metabolic adaptations to hypoxemia and suggest that targeting these pathways may offer therapeutic options to mitigate the endocrine and metabolic impairments observed in FGR fetuses.

### The impact of heat stress-induced placental insufficiency on fetal muscle fiber myogenesis and proliferation

In livestock species, myogenesis occurs during embryonic and fetal development via satellite cell proliferation, differentiation, and fusion of myoblasts into multinucleated myofibers.^67,68^ The absolute fiber number is established around the time of birth. Postnatal muscle regeneration and growth involve activation of quiescent satellite cells that reside between the sarcolemma and the basement membrane of myofibers. These quiescent satellite cells are activated only during hypertrophic growth and repair in postnatal skeletal muscle by fusing into existing muscle fibers.^69^ Although different animal species have distinct developmental timelines and rates of fetal myogenesis, the formation of skeletal muscle in livestock is characterized in a biphasic manner.^67,70^ The formation of primary muscle fibers starts from early to mid-gestation, followed by the formation of secondary and tertiary muscle fibers. For example, in sheep, the formation of primary muscle fibers starts at approximately day 30 (20 %) of gestation, whereas most secondary muscle fibers are formed at approximately day 85 (60 %) of gestation.^71,72^

In the hindlimb of near-term fetal sheep, skeletal muscle constitutes approximately 40% of the total mass and exhibits both slow- and fast-twitch fibers, similar to other major muscle groups in the body.^73,74^ This similarity in muscle structure and composition makes the hindlimb an effective and representative sample for studies on muscle physiology. Hindlimb muscle from near-term fetal sheep exposed to maternal HS has fewer total myofibers and BrdU-positive myonuclei, indicating reduced myogenesis and fewer proliferating satellite cells.^75^ Similarly, in fetal piglets, a lower density of primary muscle fibers was observed as early as mid-gestation due to maternal HS.^76^ Impaired myogenesis in FGR muscle is likely driven by decreased proliferative capacity of muscle satellite cells as the formation of new myofibers requires the fusion of activated satellite cells into myotubes.^69^ Studies have shown that hindlimb muscle from FGR fetuses proliferated at slower rates and expressed lower levels of cell cycle-related genes,^34,77,78^ resulting in fewer satellite cells and reduced DNA synthesis rates in FGR muscle.^79,80^

To further investigate cellular adaptation in fetal skeletal muscle, satellite cells (myoblasts) can be isolated from the hindlimb.^81^ When myoblasts were cultured for 3 d in growth media supplemented with serum collected from FGR fetuses (hormone-limited media), FGR myoblasts showed slower proliferation rates than control myoblasts.^77^ Furthermore, when FGR myoblasts were cultured with serum from control fetuses, their proliferation rates increased but remained slower than controls with control serum.^77^ In another study, when isolated myoblasts were cultured in nutrient-enriched media and stimulated with insulin for 5 d, FGR myoblasts had normal or higher proliferative rates.^34^ These findings demonstrate that FGR myoblasts have intrinsic deficiencies in proliferation but remain responsive to sustained extrinsic anabolic stimulation. These observations also indicate that the reduced proliferation capacity in FGR myoblasts is, at least partially, reversible, highlighting the potential for nutrient or hormone supplementation to support muscle growth, as discussed in later sections.

### The impact of heat stress-induced placental insufficiency on fetal muscle hypertrophy and protein synthesis

After myogenesis concludes, muscle fibers continue to grow through increases in diameter and length, a process known as hypertrophy. Muscle hypertrophy is driven by continued satellite cell activation to proliferate and fuse with established multinucleated myotubes during late fetal and postnatal life.^69^ Increases in DNA and myonuclei content contribute to increased muscle protein accretion. Skeletal muscle net protein accretion is associated with the balance between protein synthesis and degradation. When protein synthesis occurs at a faster rate than protein degradation, it leads to net protein accretion and myofiber hypertrophy.

Protein synthesis is stimulated by the PI3K/AKT/mTORC1 signaling pathway in response to anabolic nutrients and/or growth factors.^82–84^ Under conditions of adequate nutrients, growth factors such as insulin and IGF-1 bind to their respective tyrosine kinase receptors on the surface of a myocyte. The binding activity phosphorylates the receptor and PI3K/Akt kinases, which activates mTOR via inhibition of the tuberous sclerosis complex 2 (TSC2).^85^ The activation of mTORC1 then promotes the dissociation of 4E-binding protein 1 (4EBP1) from the eukaryotic translation initiation factor 4E (eIF4E) complex, enabling the formation of the active eIF4F translation complex and thus increasing protein synthesis.^86^ Conversely, activated mTORC1 inhibits protein degradation by blocking the autophagy-lysosome pathway.^87^ Under nutrient-restricted conditions, hypoxia, low energy availability (elevated AMP:ATP ratio), and reduced growth factor concentrations activate AMP-activated protein kinase (AMPK) signaling, often through HIF-1-dependent pathways. This activation suppresses mTORC1 activity, thereby inhibiting protein synthesis.^88,89^ However, it is important to note that the specific role of AMPK in skeletal muscle of FGR fetuses remains uncertain. For instance, no significant differences in phosphorylated or total AMPK protein abundance were observed in FGR fetal muscle.^28,90^ While this does not eliminate the possibility of AMPK involvement, further research is needed to determine whether these pathways are consistently activated in the muscle tissue of FGR fetuses exposed to HS-induced placental insufficiency.

Hindlimb muscles from FGR fetal sheep near term had uniformly smaller myofibers compared to normally grown fetuses, regardless of fiber type.^74^ Additionally, protein synthesis and accretion rates were 36 and 55% lower, respectively.^35^ This reduction in muscle net protein accretion explains the slower growth rates observed in FGR fetuses.^35,91^ To elucidate protein metabolism in developing fetuses, isotopically labeled essential amino acid tracer techniques were used.^29,92–94^ These techniques allow for the simultaneous quantification of net protein synthesis and breakdown in the whole body or in the hindlimb specifically. Using tracer methodologies, studies have demonstrated reduced whole-body protein accretion in fetal sheep exposed to hypoxemia,^93,95^ hypoglycemia,^92^ and elevated circulating cortisol or norepinephrine concentrations,^96,97^ all of which are associated with the internal FGR fetal milieu.^98^ These studies demonstrate that the causes of slower muscle growth in FGR fetuses are multifaceted. While decreased protein synthesis is evident in FGR fetal sheep, it remains less certain whether muscle protein degradation is upregulated. We have recently shown increased expression of transcripts associated with the autophagic/lysosomal proteolysis pathway (e.g., LC3B, BNIP3L, and GABARAPL1) in FGR fetal sheep muscle.^78^ However, others have shown that total fetal or hindlimb muscle protein breakdown rates were unaffected in HS-induced ^35^ or isovolemic hemodilution-induced FGR fetuses.^99^ Despite these findings, it is important to consider that protein synthesis and degradation rates are not mutually exclusive processes. Reduced nutrient availability due to placental insufficiency can limit protein synthesis potential as discussed above, while cellular stress may activate autophagy and protein breakdown signaling pathways in skeletal muscle.^78^

### The impacts of heat stress-induced placental insufficiency on fetal skeletal muscle energy metabolism

Interestingly, under basal conditions, late gestation FGR fetal sheep (approximately 130 dGA) exhibited near-normal rates of weight-specific hindlimb glucose uptake^35^ and whole-body glucose utilization,^26,39^ despite a reduction of approximately 30% in rates of net umbilical (fetal) glucose uptake.^25,26,35,37,39^ Given that basal and glucose-stimulated insulin concentrations were lower in FGR fetuses, near-normal rates of hindlimb glucose uptake and whole-body glucose utilization indicated increased peripheral insulin sensitivity.^26,28,100^ The discrepancy between rates of umbilical glucose uptake and fetal glucose utilization indicates that there was endogenous hepatic glucose production in FGR fetuses, which was not affected by hyperinsulinemia and demonstrated central insulin resistance.^100^ These findings reveal systemic adjustments in glucose and insulin homeostasis and signify potential reprogramming of skeletal muscle metabolism to facilitate glucose clearance and production rates in FGR fetuses.

Despite normal fetal glucose utilization rates, the fraction of glucose oxidized to carbon dioxide was less in FGR fetuses.^26,31,39^ Impaired oxidative metabolism in FGR fetuses is believed to be associated with mitochondrial dysfunction, especially in skeletal muscle.^101^ The metabolic intermediate acetyl-CoA produced from glucose (pyruvate), amino acids, and fatty acids converges in the tricarboxylic acid (TCA) cycle. The series of enzymatic reactions in the TCA cycle releases carbon dioxide and transfers electrons to energy carriers, NADH and FADH2.^102^ These reducing equivalents are subsequently oxidized at the mitochondrial electron transport chain (ETC) to produce ATP. Thus, cellular respiration via the ETC is a vital process of mitochondrial function.^103^ Using an *ex vivo* fiber optic fluorescence system, we have demonstrated a lower glucose-derived mitochondrial oxygen consumption rate (OCR) in the hindlimb muscle of FGR fetal sheep.^38,78^ Although another study reported preserved mitochondrial OCR in permeabilized muscle fibers from FGR fetuses, it also found reductions in type I oxidative fiber expression, citrate synthase (CS) activity, and mitochondrial complex I subunit expression, indicating mitochondrial dysfunction.^104^ Consistent with these observations, ATP content was reduced in FGR fetal muscle.^90,104^ In response to diminished ATP availability, ATP expenditure was downregulated, as evidenced by reduced activity of ATP-dependent Na+/K+ ATPase in FGR fetal muscle.^90^ This coordinated reduction in ATP availability and expenditure represents an adaptive response that conserves energy under conditions of limited substrate availability but restricts energy-intensive processes essential for normal muscle growth.

**Figure 2.**
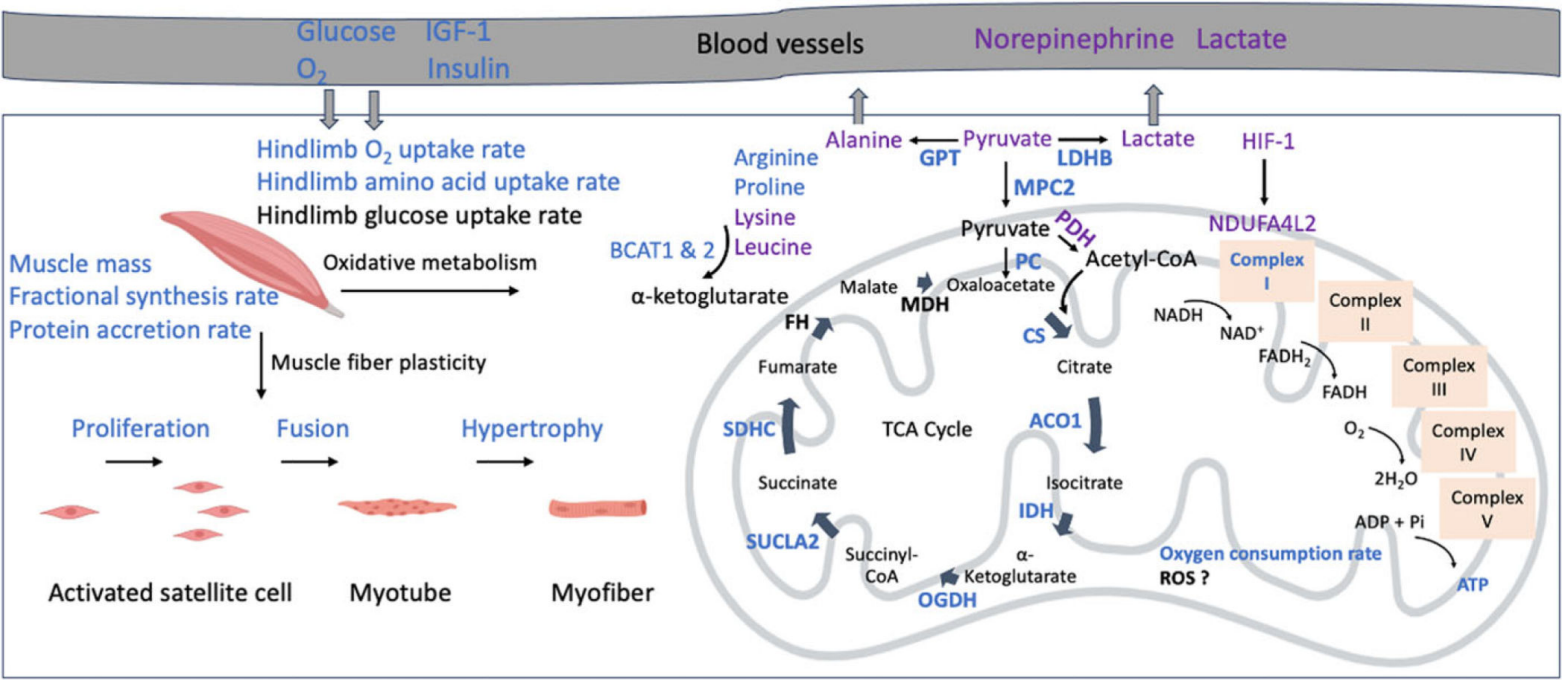
Summary of fetal hindlimb skeletal muscle adaptations in heat stress-induced placental insufficiency and fetal growth restriction (FGR) in sheep. The upregulated and downregulated (FGR compared to control) metabolic pathways and substrate concentrations are indicated in purple and blue text, respectively, whereas the descriptive labels and unchanged events are shown in black text. FGR fetuses had lower circulating nutrient and anabolic hormone concentrations while having higher norepinephrine and lactate concentrations. Hindlimb muscle from FGR fetuses had lower satellite cell myogenesis and hypertrophy, contributing to lower muscle mass and protein accretion. There was reduced pyruvate-driven oxidation in the mitochondria of FGR fetuses, leading to the accumulation of intramuscular pyruvate. The impaired pyruvate oxidation capacity was associated with a combination of mitochondrial electron transport chain dysfunction (e.g., upregulation of NDUFA4L2) and inhibited pyruvate flux into the mitochondrial matrix and its conversion into the TCA cycle (e.g., downregulation of MPC2, PC, and CS). Additionally, there was reduced expression of TCA-related enzymes. These mitochondrial deficits were associated with lower mitochondrial oxygen consumption rates and reduced ATP production. HIF-1, hypoxia-inducible factor 1; NDUFA4L2, NADH dehydrogenase (ubiquinone) 1 α subcomplex, 4-like 2; GPT, glutamic-pyruvic transaminase (alanine aminotransferase); LDHB, lactate dehydrogenase B; MPC2, mitochondrial pyruvate carrier 2; PC, pyruvate carboxylase; PDH, pyruvate dehydrogenase; CS, citrate synthase; ACO1, aconitase 1; IDH, isocitrate dehydrogenase; OGDH, oxoglutarate dehydrogenase; SUCLA2, succinate-CoA ligase ADP-forming subunit b; SDHC, succinate dehydrogenase subunit C; FH, fumarate hydratase; MDH, malate dehydrogenase; BCAT1 and 2, branched-chain amino acid transaminase 1 and 2.

Experimental evidence has shown that reduced glucose oxidation in FGR fetuses is associated with a combination of mitochondrial ETC dysfunction and inhibition of pyruvate flux into the mitochondrial matrix (Fig. 2). Biceps femoris muscle from FGR fetal sheep had lower protein abundance of subunit (NDUFB8) and reduced activity of mitochondrial complex I, the largest component of the respiratory chain.^38,104^ We also showed that FGR fetal muscle had a robust increase in mRNA and protein expression of NADH dehydrogenase 1 α subcomplex 4-like 2 (NDUFA4L2),^38,78^ a negative regulator of mitochondrial complex I activity. During hypoxia, NDUFA4L2 is upregulated by hypoxia-inducible factor 1a (HIF1a) and integrates into mitochondrial complex I and reduces the activity of the ETC^105^. The induction of NDUFA4L2 also partially explains reduced mitochondrial OCRs in cancer cells under hypoxic conditions.^105^ In murine skeletal muscle, ectopic overexpression of NDUFA4L2 reduced mitochondrial respiration and caused *a* ~ 20% reduction in muscle mass.^106^ Thus, we hypothesize that hypoxic induction of mitochondrial NDUFA4L2 decreases complex I activity, which contributes to lower OCRs and further defines reduced muscle growth in FGR fetal muscle (Fig. 2).

In addition to mitochondrial ETC dysfunction, studies have shown downregulation of mitochondrial enzymes (e.g., pyruvate carboxylase (PC), CS, and pyruvate carrier 2 (MPC2)) that govern pyruvate’s flux into the mitochondrial matrix and conversion into the TCA cycle in FGR fetal muscle (Fig. 2).^38,78,104,107^ Despite lower mRNA expression of pyruvate dehydrogenase(PDH),^33^ which converts pyruvate to acetyl-CoA, there was increased PDH enzyme activity in FGR muscle.^107^ The greater PDH activity indicates a compensatory mechanism in response to deficiencies in pyruvate transport into the mitochondria to enter the TCA cycle. However, even with enhanced PDH activity, pyruvate-specific mitochondrial oxidation remained impaired, as evidenced by reduced mitochondrial OCR in permeabilized soleus muscle fibers from FGR fetuses.^78^ These findings suggest that mitochondrial entry of pyruvate may still be restricted, possibly due to downregulation of MPC2 or PC, leading to intramuscular pyruvate accumulation. In turn, excess pyruvate appears to be diverted to alternative pathways, such as transamination to alanine (Fig. 2).^33^

Placental insufficiency adversely affects hindlimb muscle amino acid metabolism. Weight-specific net uptake rates for nearly all essential (e.g., lysine, threonine, and phenylalanine) and non-essential (e.g., alanine, arginine, glycine, and glutamate) amino acids in the hindlimb of FGR fetal sheep were reduced.^33,35^ The lower amino acid uptake rate in FGR hindlimb correlated with reduced hindlimb size and muscle mass, indicating that diminished amino acid metabolism contributes to impaired muscle growth. Alongside reduced amino acid uptake, the expression of branched-chain amino acid transaminase 1 (BCAT1) and BCAT2, key enzymes responsible for branched-chain amino acid (BCAA) transamination, was significantly reduced in FGR muscle.^33^ This may have contributed to higher intramuscular concentrations of BCAA (e.g., lysine and isoleucine) and reduced utilization of nitrogen from BCAA catabolism (Fig. 2). It is proposed that FGR muscle shifted amino acid metabolism away from protein accretion and toward the production and release of the ammoniagenic amino acids, including alanine, glycine, and glutamine, into the circulation.^33^ These metabolic adaptations are likely mechanisms to conserve energy and maintain nitrogen balance under nutrient-stressed conditions, though at the cost of muscle protein synthesis and growth.

### Strategies to improve muscle growth in growth-restricted animals

Intervention strategies are limited to attenuate the HS impact on gestating animals and fetal development, especially to improve placental function and fetal skeletal muscle growth. While cooling technologies, such as fans, misters, conductive cooling, and shade structures, have shown promising results in improving reproductive performance in livestock species,^108–110^ their implementation is often costly, energy-intensive, and limited to certain environments or facilities. Recent studies identifying genetic markers and pathways associated with improved heat tolerance and adaptive traits in HS pregnant ewes offer a complementary approach.^111,112^ Research over the past two decades has only begun to explore targeted nutritional or hormonal strategies to improve birth outcomes for growth-restricted fetuses. A critical initial step involves understanding how externally supplied nutrients are transferred to the fetus and how the fetus metabolically responds to these nutritional inputs. To address these challenges, research has focused on the effect of direct fetal nutrient delivery on fetal metabolism, hormone profiles, and growth. Here, we will summarize the current understanding of targeted fetal nutritional supplementation on fetal and muscle growth, primarily informed by studies on chronically catheterized normal or FGR fetal sheep. Although direct fetal infusions are not practical in farm practice or in human pregnancies, insights from these experiments could inform optimized maternal nutritional strategies to mitigate the adverse effects of HS on fetal and postnatal development.

#### IGF-1

Insulin-like growth factor-1 (IGF-1) promotes fetal growth, and its circulating concentrations are lower in FGR fetuses.^29,30,35^ *In vitro*, IGF-1 promoted both myoblast proliferation and differentiation by activation of PI3K/AKT/mTOR pathways.^113,114^ In fetal sheep, acute (3–7 h) intravenous infusion of IGF-1 increased hindlimb blood flow, and skeletal muscle protein synthesis and accretion rates.^115,116^ However, the acute anabolic effect of IGF-1 on muscle protein synthesis diminished when IGF-1 was administered chronically (7 d) into normal sheep fetuses, although myoblast proliferation rates were increased.^117^ Interestingly, the IGF-1 infusion lowered fetal glucose and insulin concentrations,^115,117^ which impaired β-cell function.^118^ As insulin is another potent stimulator of protein synthesis, it has been proposed that maintaining circulating fetal insulin (euinsulinemia), as well as glucose (euglycemia) and amino acid (euaminoacidemia) concentrations during fetal IGF-1 infusion is necessary to maximally support body growth. A recent study demonstrated that when fetal glucose and insulin concentrations were maintained, fetal chronic infusion of IGF-1 during late gestation improved fetal body weights by 23% in normally grown fetal sheep.^119^ In maternal uterine artery embolization induced FGR fetal sheep, intra-amniotic IGF-1 injections at mid- to late-gestation increased growth rates and partially restored fetal body weights.^120,121^ Similarly, a weekly intra-amniotic injection of recombinant human IGF-1 for 1 month during mid-gestation enhanced carcass lean tissue and decreased abdominal adipose tissue deposition in FGR juvenile lambs.^122^ However, in FGR fetal sheep, a 1-week IGF-1 analog infusion did not improve body growth, but circulating fetal amino acid concentrations decreased during the infusion.^123^

#### Insulin

Hypoinsulinemia is a hallmark in FGR fetuses due to their worsening hypoglycemia, hypoxemia, and impaired pancreatic β-cell function.^26,28,61^ Insulin activates the PI3K/Akt/mTOR pathway and protein synthesis.^83,84,124^ In addition, insulin also stimulates glucose uptake, providing energy for muscle cell metabolism. Hyperinsulinemic-euglycemic-euaminoacidemic clamping techniques showed that acute (5–7 h) hyperinsulinemia increased skeletal muscle protein accretion rates in fetal sheep^125^ and neonatal piglets.^126,127^ When infused chronically (1–2 weeks) at 75% of gestation, insulin only increased skeletal muscle myoblast proliferation rates in both normally grown and FGR fetal sheep without affecting muscle cell differentiation, myofiber number, hypertrophy, or protein synthesis rates.^32,128^ Three reasons were postulated that may explain the absence of increased protein synthesis with the chronic insulin infusion in fetal sheep. First, administration of insulin decreased circulating fetal essential amino acid concentrations without the compensatory upregulation of fetal amino acid uptake rates from the placenta^32^. Second, both insulin and IGF-1 infusions did not alleviate fetal hypoxemia and even further decreased fetal blood oxygen content.^115,119,125,128^ Finally, insulin infusions increased norepinephrine concentrations, possibly associated with reduced blood oxygen content.^32,129^ Lower amino acid and oxygen and higher norepinephrine concentrations all limit muscle protein synthesis in the fetus.^29,93,97,130^ Further studies are needed to explore the long-term benefits of fetal insulin supplementation while maintaining or improving fetal amino acid, oxygenation, and/or fetal IGF-1 concentrations.

#### Fetal oxygenation

**Figure 3.**
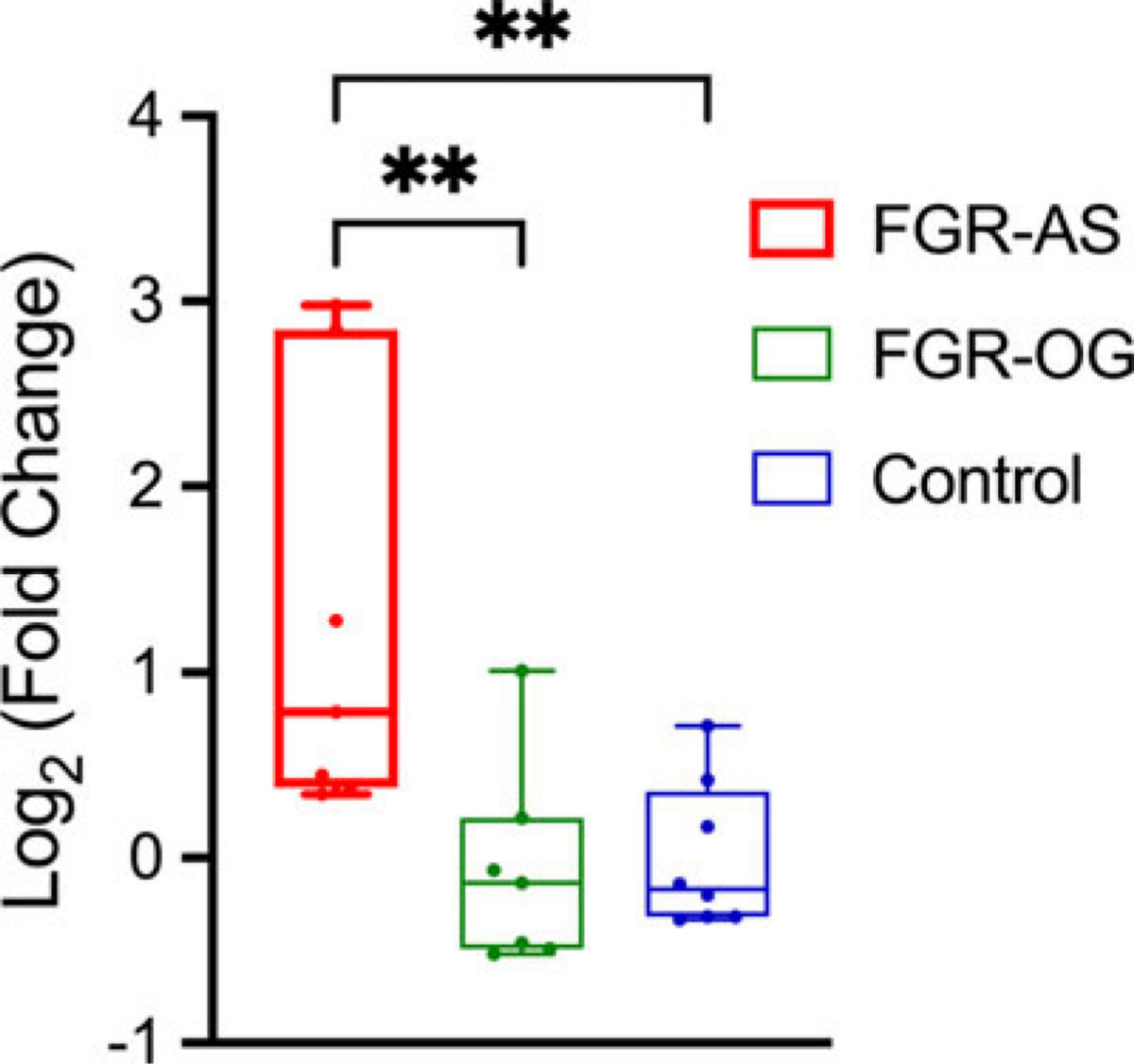
Fetal oxygen and glucose treatment for 5 d normalizes NDUFA4L2 expression in heat stress-induced placental insufficiency and fetal growth restricted (FGR) muscle. Expression levels of NADH dehydrogenase 1 α subcomplex 4-like 2 (NDUFA4L2) mRNA were determined in the biceps femoris muscle of FGR-air and saline (FGR-AS; *n* = 7), FGR-oxygen and glucose (FGR-OG; *n* = 7), and control (CON; *n* = 8) fetuses. Quantitative polymerase chain reaction (PCR) results are presented as the log_2_ fold change. Each data point represents the value from an individual fetus within its respective experimental group. Box plots show the interquartile range and median (horizontal line), with whiskers indicating minimum and maximum values. Groups were analyzed with an ANOVA. **denotes *P* < 0.01 differences between groups. The figure is based on unpublished experimental data.

Fetal hypoxemia alone causes FGR in farm animals.^131–134^ Thus, restoration of fetal oxygenation remains an attractive therapeutic strategy to improve the growth of FGR fetuses. To raise fetal oxygenation, maternal oxygen supplementation is commonly applied because oxygen can diffuse across the concentration gradient through the placenta. We have demonstrated that maternal tracheal insufflation of humidified oxygen during late gestation increased fetal oxygenation in FGR fetal sheep.^39,66^ In addition, fetal oxygenation restored GSIS in both fetal^66^ and neonatal^135^ FGR lambs. At neonatal stages, FGR lambs from heat-stressed ewes that received intermittent oxygen therapy for 2 weeks during late gestation had larger birth weights, neonatal growth rates, and hindlimb mass at 1 month of age compared to untreated FGR lambs.^135^ However, it was noted that acute fetal oxygenation alone neither lowered fetal plasma catecholamine concentrations nor attenuated fetal hypoglycemia in FGR fetuses.^66^ Thus, we postulate that other anabolic stimuli, coupled with fetal oxygenation, should be considered to fully restore the pathologies of FGR. We have recently demonstrated that maternal tracheal insufflation of humidified oxygen combined with fetal glucose infusion for 5 d at late gestation normalized fetal oxygen, glucose, and insulin concentrations in FGR fetal sheep.^39^ In skeletal muscle, the mRNA expression of NDUFA4L2 was increased 3-fold in FGR fetuses, and this upregulation was attenuated by chronic fetal treatment with oxygen and glucose (Fig. 3). Given the negative association between NDUFA4L2 and mitochondrial complex I activity in hypoxia,^105^ the normalization of NDUFA4L2 indicates improvement in fetal oxygenation and mitochondrial respiratory function in FGR muscle. Further studies are needed to investigate whether oxygen and glucose administration is sufficient to improve the oxidative metabolism of glucose and spare amino acids for tissue accretion in skeletal muscle.

#### Amino acids

Placental insufficiency is associated with decreased umbilical and hindlimb uptake rates for amino acids.^35,53^ The amino acid deficiency can be mitigated by exogenous amino acid supplementation.^136^ Acute (3–4 h) infusions of mixed amino acids into FGR fetal sheep increased net fetal protein accretion rates, largely due to inhibition of fetal whole body protein breakdown.^29,137^ However, prolonged (9–12 d) infusions of mixed amino acids or leucine alone to FGR and normally growing fetuses have had limited impacts on whole-body protein synthesis or breakdown, despite increasing plasma amino acid concentrations and leucine oxidation rates.^36,138,139^

Similar to the effects of insulin and IGF-1 infusions, neither hypercatecholaminemia nor hypoxemia was improved by chronic fetal amino acid infusions.^36^ Therefore, adequate fetal oxygenation may be necessary to maximize the benefits of amino acid supplementation in FGR fetuses. Alternatively, amino acid availability may not be a limiting factor compromising protein synthesis in FGR fetuses. There were normal or above normal plasma concentrations for most amino acids in FGR fetuses, despite impaired umbilical net uptake for amino acids.^35,53^ Instead, increased amino acid oxidation and/or higher efflux of the gluconeogenic amino acids for hepatic gluconeogenesis may limit the use of amino acids for protein synthesis in the muscle under hypoglycemic conditions.^28,92,140^ Further research is needed to determine whether fetal oxygenation and increased glucose availability will enhance the utilization of amino acids for protein synthesis.

### Other prenatal nutritional strategies

Increasing evidence suggests that oxidative stress and inflammation contribute to the pathogenesis of FGR, supporting the potential of antioxidant and anti-inflammatory therapies as intervention strategies.^134,141,142^ Maternal or fetal antioxidant treatments, such as resveratrol,^143^ melatonin,^144–146^ and vitamin C^144^ improved uteroplacental blood flow in pregnant sheep or cattle with normal pregnancies. In compromised pregnancies, maternal antioxidant supplementation improved fetal growth or birth weight in livestock species.^142,147–150^ In addition to oxidative stress, studies also demonstrated enhanced fetal systemic or muscle-specific inflammatory cytokine signaling in compromised pregnancies (e.g., maternal HS or inflammation).^151–153^ Daily fetal infusion of anti-inflammatory ω−3 polyunsaturated fatty acids for 5 d during late gestation alleviated fetal hypoglycemia and hypoxemia and recovered hindlimb muscle growth in HS-induced FGR fetal sheep.^154,155^

### Challenges to restore growth performance in FGR offspring

It remains a major challenge to fully restore the growth of FGR offspring. Most of the abovementioned studies attempted nutrient, oxygen, and hormone replacement strategies at around 90% of gestation, or near term. It may be that toward the end of gestation, adaptations to placental insufficiency become fixed and difficult to reverse, requiring interventions earlier in gestation. After birth, FGR neonatal animals may continue to demonstrate limitations in the capacity to exhibit normal trajectories of postnatal growth and additionally have more difficulties ingesting sufficient colostrum or milk due to their lower vigor. Thus, they have lower energy reserves that affect survival and growth.^156^ Low-birth-weight animals are commonly provided with compensatory nutrition to promote catch-up growth.^157–160^ However, this intervention can increase the risks of metabolic syndrome in the offspring, which can manifest as excess fat deposition,^161,162^ abnormal immune function,^163^ and muscle mitochondrial dysfunction.^164^ Therefore, it is essential to investigate the mechanisms behind how both FGR fetuses and neonates respond to nutrient stimuli and the long-term consequences. For instance, FGR offspring often exhibit impaired gastrointestinal development, which can limit nutrient absorption and digestibility, as observed in pigs^165–168^ and lambs.^169–171^ Additionally, the intrauterine environment may also program skeletal muscle anabolic resistance, as indicated by blunted insulin- and amino acid-induced muscle protein synthesis in preterm neonatal piglets.^172,173^ Thus, optimizing nutrient absorption efficiency while avoiding the metabolic dysfunctions commonly associated with excessive catch-up growth is crucial and warrants further investigation when considering nutritional strategies for FGR neonates.

## Conclusion

The research reviewed highlights the significant impact of placental insufficiency, particularly induced by maternal HS, on fetal growth and skeletal muscle development. Studies in HS-induced placental insufficiency and FGR fetal sheep have demonstrated lower rates of net umbilical uptake for oxygen, glucose, and amino acids. The reductions in anabolic substrate availability, coupled with hypoxia, limit fetal skeletal muscle growth. HS-induced placental insufficiency disrupts fetal muscle satellite cell proliferation, which lowers myogenesis and myofiber hypertrophy. Muscle fiber deficiencies are associated with reduced protein synthesis rates and muscle mass, with long-lasting consequences that extend into postnatal life. Mechanisms underlying fetal muscle growth restriction are believed to be partially due to impaired energy metabolism in the mitochondria, associated with the combination of mitochondrial ETC dysfunction and reduction of pyruvate flux into the mitochondrial matrix. We postulate that mitochondrial complex I subunit NDUFA4L2 is a major regulator of these events. Over recent decades, research has begun to explore nutritional strategies aimed at improving fetal and skeletal muscle growth in FGR animals. Despite these efforts, few effective approaches remain for enhancing whole-body or skeletal muscle-specific growth in FGR fetuses, particularly when relying on the supplementation of a single nutrient substrate. Continued research is essential to develop nutritional and management strategies that can simultaneously address fetal oxygenation while maintaining the metabolic requirements of key nutrient substrates such as glucose and amino acids. While direct fetal infusions of nutrients are not practical in agricultural settings and human pregnancies, these approaches are invaluable in understanding how FGR fetuses respond to external nutrient supply. By assessing fetal hormonal profiles, protein metabolism, and growth in response to these interventions, we can better understand the potential for reversing or mitigating the effects of placental insufficiency.
